# *TFEB*-Amplified Renal Cell Carcinoma: Integrating Histopathologic and Molecular Diagnostics with Therapeutic Implications

**DOI:** 10.1177/10668969251379897

**Published:** 2025-10-03

**Authors:** Julia Esber, Bibianna M. Purgina, Kevin Hogan, Nicola Schieda, Finn M. Auld, Ilias Cagiannos, Trevor A. Flood

**Affiliations:** 1 Faculty of Science, 12365 University of Ottawa, Ottawa, ON, Canada; 2 Division of Anatomic Pathology, 574446 The Ottawa Hospital / University of Ottawa, Ottawa, ON, Canada; 3 Department of Radiology, 27337 The Ottawa Hospital/University of Ottawa, Ottawa, ON, Canada; 4 Division of Urology, 27337 The Ottawa Hospital/University of Ottawa, Ottawa, ON, Canada

**Keywords:** urologic neoplasms, renal cell carcinoma, *TFEB*-amplified renal cell carcinoma, gene amplification, immunohistochemistry, fluorescent in situ hybridization (FISH), angiogenesis inhibitors, immunotherapy, nephrectomy, neoplasm metastasis

## Abstract

*TFEB*-amplified renal cell carcinoma (RCC) represents a newly described and rare aggressive molecular subtype of RCC that is characterized by *TFEB* gene amplification (6p21.1). These tumors are characterized by heterogeneous morphology (papillary, tubulocystic, and nested patterns), eosinophilic/clear cytoplasm, and prominent nucleoli with perinucleolar clearing. We present a diagnostically challenging lesion in a 72-year-old man who was found to have a 9.8 cm partially necrotic renal mass during work up for decreased renal function and hematuria. The nephrectomy specimen revealed a large tumor invading into the renal vein, renal sinus, and perinephric fat. Histology showed a heterogeneous tumor of clear or eosinophilic cells with prominent nucleoli and occasional perinucleolar clearing. Apically oriented nuclei were noted surrounding tubulocystic structures, as were several multinucleated tumor cells. Immunohistochemistry demonstrated Pan-keratin/Mel-A positivity. Notably, PAX8 was negative in the majority of the tumor and PD-L1 had a CPS of <5. Fluorescent in situ hybridization showed *TFEB* amplification (6-15 copies) and confirmed the diagnosis of *TFEB*-amplified RCC. Despite combined immunotherapy and anti-angiogenic therapy, the patient succumbed to rapid metastatic progression 5 months postoperatively, underscoring the tumor's aggressive behavior and current therapeutic limitations. This report outlines the diagnostic complexities associated with *TFEB*-amplified RCC and emphasizes the need for effective management strategies.

## Introduction

Renal cell carcinoma (RCC) classification is increasingly relying on incorporation of molecular signatures to distinguish between subtypes and characterize emerging entities. Among these, the *TFEB*-altered RCC family encompasses the *TFEB*-rearranged and *TFEB*-amplified RCCs.^1^

*TFEB*-rearranged RCCs present frequently in younger patients and are defined by chromosomal rearrangement involving the *TFEB* transcription factor gene (6p21.1) that frequently partners with *MALAT1*, resulting in aberrant transcriptional activation. These tumors have unique morphologic features characterized by a biphasic population of large cells with voluminous clear to eosinophilic cytoplasm, and small lymphocyte-like cells. The majority of these tumors occur in younger patients are associated with indolent behavior.^2^

In contrast, *TFEB*-amplified RCCs affect older patients and show amplification of the 6p21.1 locus, which drives *TFEB* overexpression and activates downstream oncogenic pathways. The tumors tend to be morphologically heterogeneous and are composed of high-grade epithelioid cells with eosinophilic cytoplasm arranged in nests, papillary, and pseudopapillary architectures.^3,4^ Unlike *TFEB*-altered RCCs, *TFEB*-amplified RCCs demonstrate aggressive behavior with frequent metastases, advanced stage at presentation, and a reduced survival rate.^5–7^

*TFEB*-amplified RCCs are rare and newly described renal malignancies and increased awareness of this entity is essential to help guide appropriate management. The findings presented herein highlight the diagnostic challenges associated with *TFEB*-amplified RCCs and underscores the necessity of molecular confirmation for definitive classification.

## Clinical Summary

**Figure 1.**
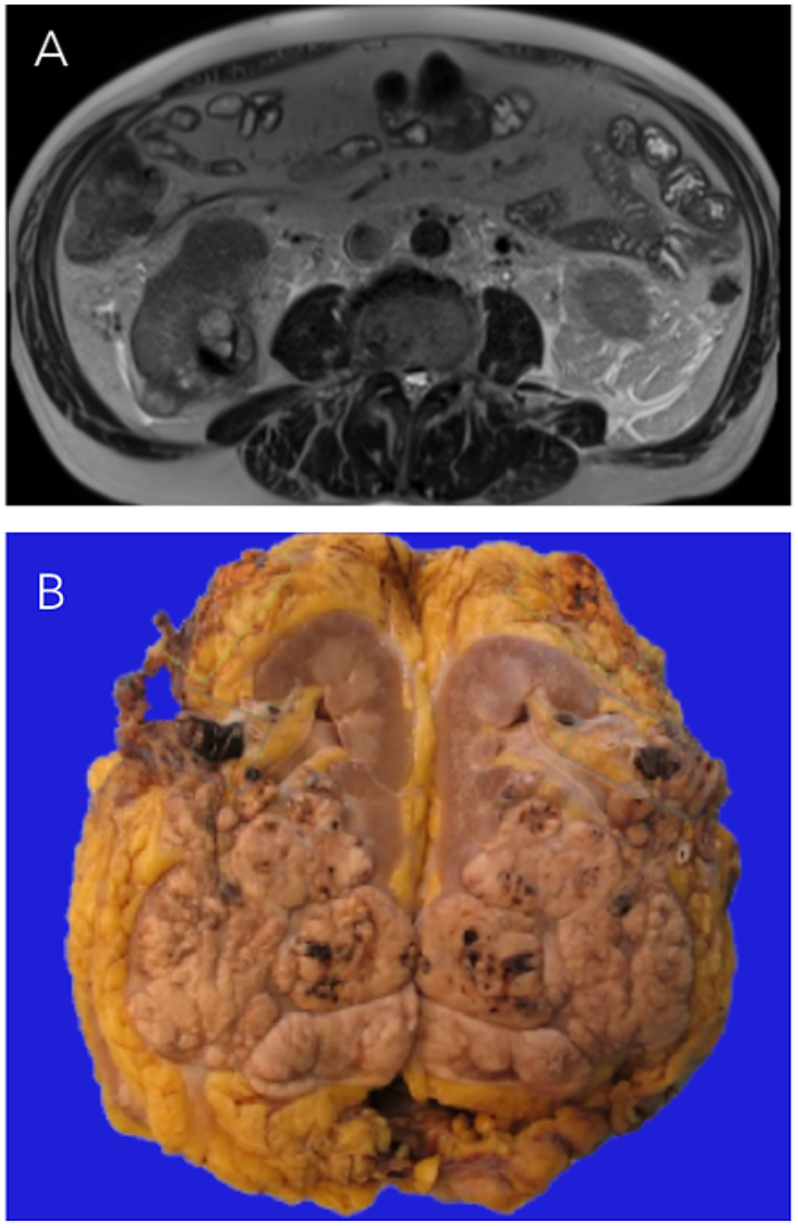
(A) Axial T2-weighted MRI image showing the infiltrative mass in the posterior lower pole of the right kidney. (B) Nephrectomy specimen showing a multinodular mass indicative of retrograde venous invasion, with tumor infiltration into the renal vein, perinephric fat, and renal sinus.

A 72-year-old man presented with worsening renal function and hematuria which prompted an abdominal ultrasound that revealed a large right-sided renal mass. CT and MRI (Figure 1A) further characterized the lesion as a 9.6 cm exophytic lower pole mass with tumor thrombus extending through the right renal vein and into the infrahepatic inferior vena cava (IVC). The patient underwent open radical nephrectomy with adrenalectomy and IVC thrombectomy. A metastatic liver deposit was identified intraoperatively and was surgically excised.

Postoperatively, the patient received two cycles of axitinib and pembrolizumab. Subsequent clinical monitoring revealed rapid elevation of liver function tests and CT abdomen/pelvis showed confluent liver metastases. Given the rapid progression of the disease despite systemic therapy, the decision was made to focus on comfort care and the patient passed away 5 months after surgery.

## Pathological Findings

Gross pathologic examination of the radical nephrectomy specimen revealed a 9.6 × 8.5 × 8.0 cm tan multinodular renal mass, suggestive of retrograde venous invasion.^8^ Tumor invaded into the perinephric adipose tissue, renal vein, and renal hilar fat (Figure 1B). Punctate foci of necrosis were observed scattered throughout the lesion. Multiple isolated tumor nodules were identified in the perinephric adipose tissue.

**Figure 2.**
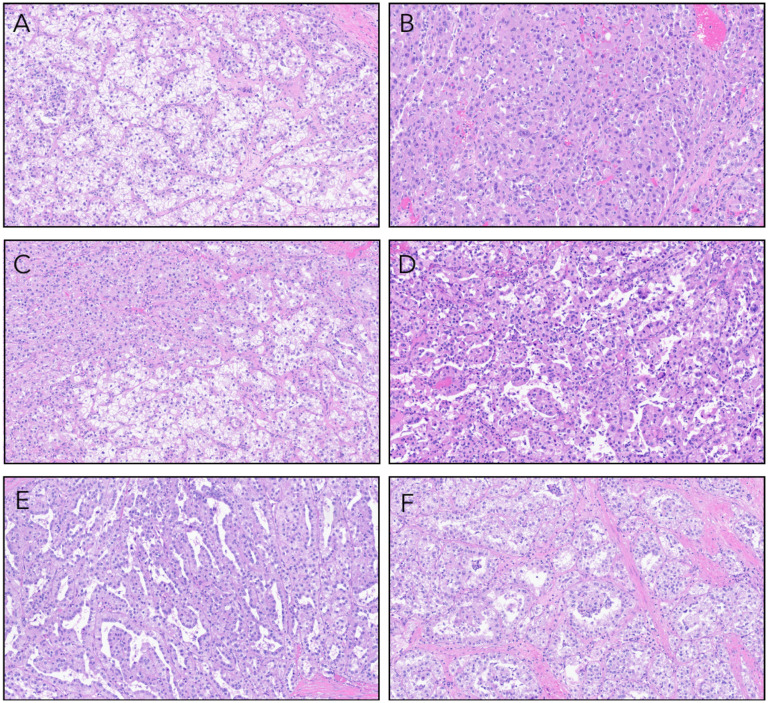
(A) Tumor composed of cells with clear cytoplasm separated into nests by thin-walled arborizing blood vessels. (B) Compact nests and sheets of high-grade eosinophilic cells. (C) A mixture of both clear and eosinophilic tumor cells. (D) Papillary and pseudopapillary projections. (E) Slit-like tubulocystic spaces lined by eosinophilic cells with apically oriented nuclei. (F) Tumor acini with clusters of malignant cells suspended within the acinar lumens.

**Figure 3.**
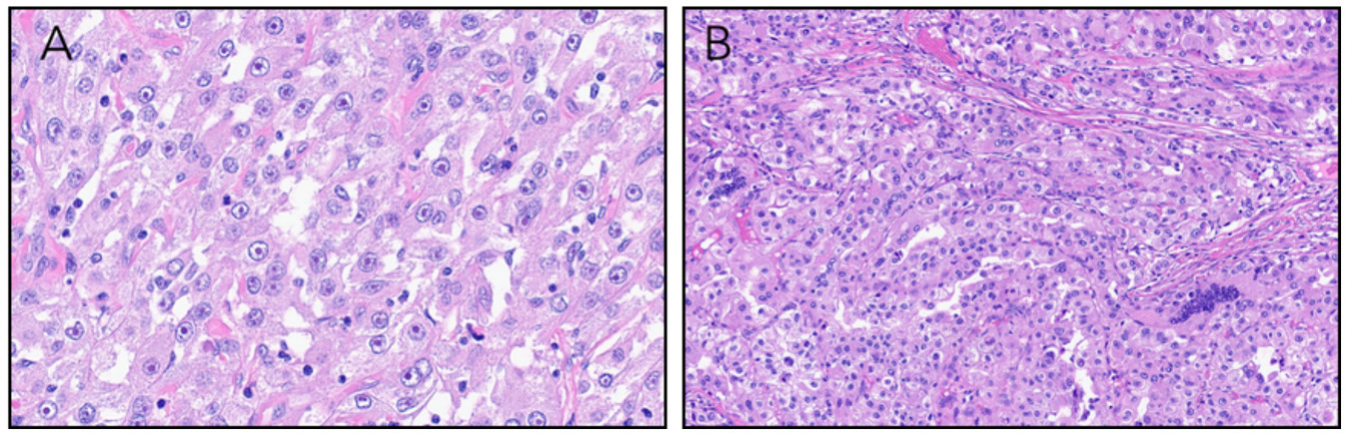
(A) Tumor cells with prominent nucleoli with surrounding perinucleolar halos. (B) Sheets of malignant cells with occasional multinucleated tumor giant cells.

Microscopically, the tumor was high grade and showed a diverse range of architectural patterns with tumor cells arranged in tubulopapillary and papillary structures, sheets, and nests. Tumor cell cytoplasm was variably clear or eosinophilic. In some areas showed apically oriented nuclei surrounding tubulopapillary lumens (Figure 2A-F). Elsewhere, nuclei featured prominent eosinophilic nucleoli with perinucleolar clearing. Multinuclear tumor giant cells were seen scattered throughout the lesion (Figure 3A-B). Adipose tissue, blood vessels with thickened walls, and psammomatous calcifications were absent.

**Figure 4.**
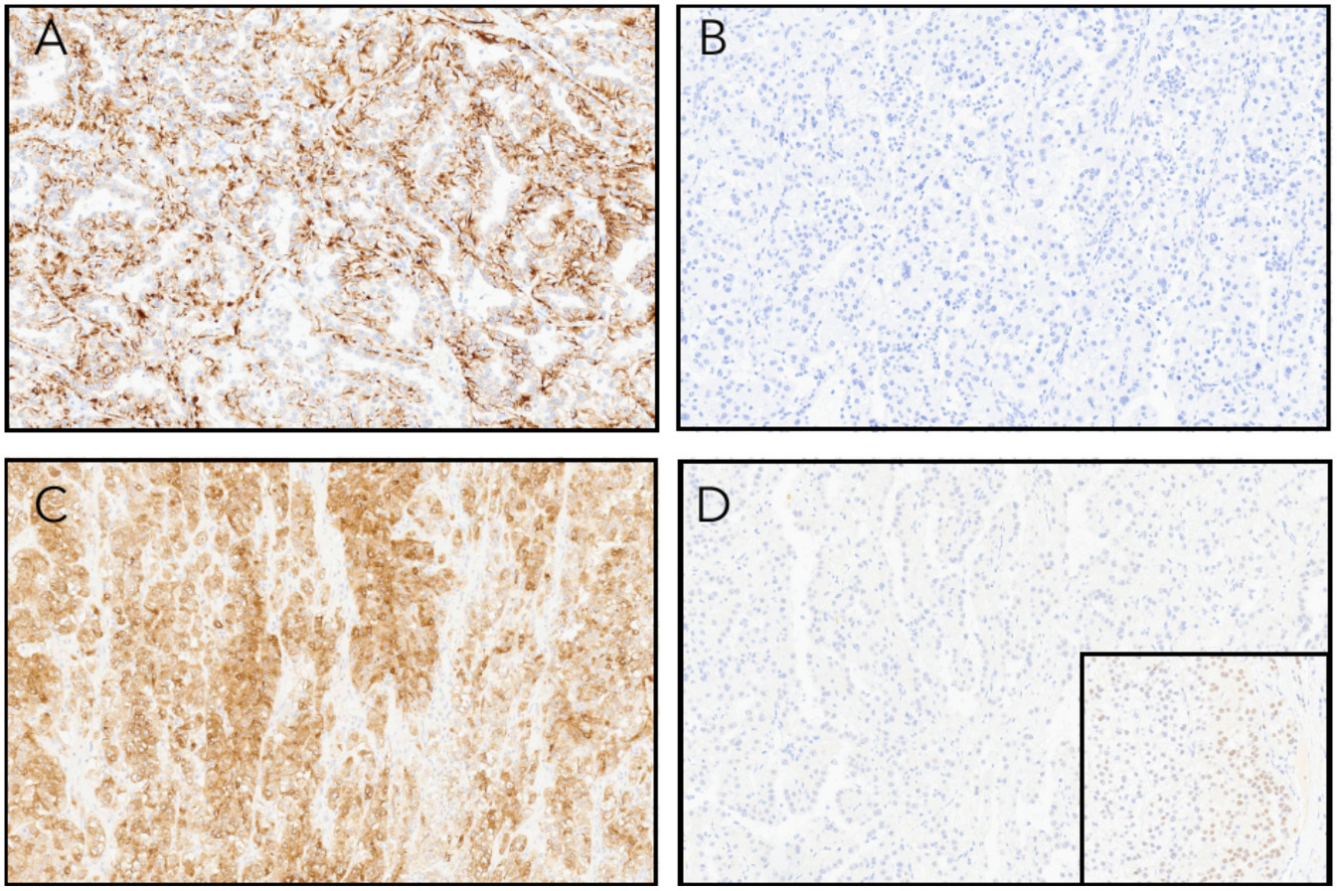
(A) Tumor cells are positive for pan-keratin immunohistochemistry. (B) Keratin 7 is negative. (C) Melan-A staining was positive although HMB45 expression was absent (not shown). (D) PAX8 was negative in the vast majority of the tumor although there were rare foci that showed weak expression (inset).

Immunohistochemistry (IHC) (Figure 4A-D) showed co-expression of pan-keratin and Melan-A. There was patchy staining with CD68 and keratin 20, while PAX8 was negative in the majority (>95%) of tumor cells. Fumarate hydratase (FH) expression was retained. The tumor cells were negative for 2SC, CD10, HMB45, racemase (AMACR), keratin 7, GATA3, S100 protein, TTF1, desmin, and SMA. MSH2, MSH6, MLH1, and PMS2 showed retained nuclear expression. PD-L1 had a CPS of <5.

**Figure 5.**
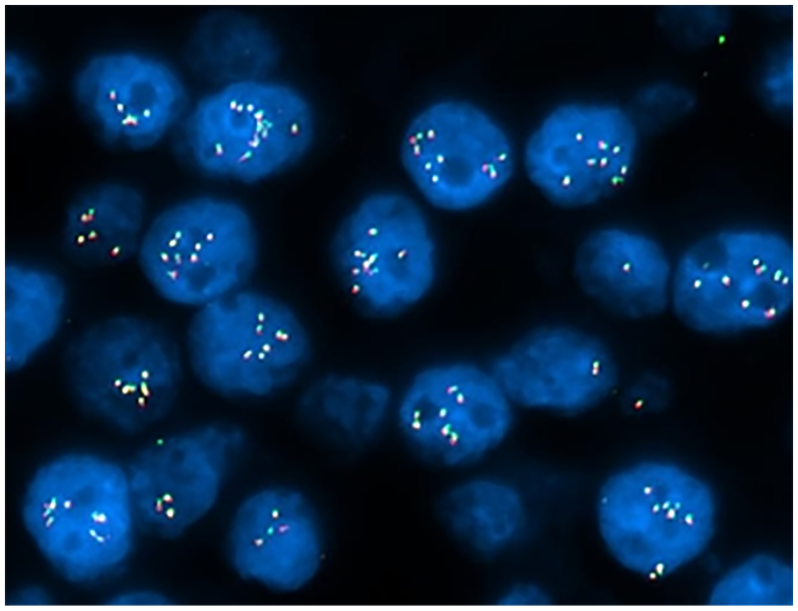
Fluorescence in situ hybridization (FISH) analysis for *TFEB* Gene amplification. Nuclei are counterstained with DAPI (blue). Red and green signals correspond to *TFEB* and control probes, respectively. Multiple fluorescent signals per nucleus indicate amplification of the *TFEB* Gene. The increased number of *TFEB* signals relative to control demonstrates gene amplification in these cells.

Fluorescent in situ hybridization (FISH) analysis was negative for rearrangement of the *TFE3* (Xp11.2) locus but showed amplification (6-15 copies) of the *TFEB* (6p21.1 locus) gene in 150/200 (75.0%) of interphase nuclei analyzed (Figure 5). Based on the cytomorphology, IHC profile, and FISH results, a diagnosis of *TFEB*-amplified RCC was made.

## Discussion

This report highlights some of the diagnostic challenges associated with *TFEB*-amplified RCC. *TFEB*-amplified RCC is a newly characterized entity with approximately 50 tumors documented in the literature,^1^ and there may be limited awareness of its morphologic spectrum. Accurate diagnosis of these neoplasms is critical given its aggressive clinical behavior and potential therapeutic implications.

The tumor described in this manuscript exhibited several architectural patterns, including pseudopapillae, true papillae, solid nests, and sheets. This is consistent with the morphological diversity described in *TFEB*-amplified RCC.^7,9^ Cytoplasmic variability was also notable, with cells displaying either clear or eosinophilic cytoplasm as previously reported.^7,10^ Notably, two distinctive and previously reported^3,7^ features were observed in this tumor: (1) tubulocystic structures lined by eosinophilic cells with apically polarized nuclei, and (2) strikingly prominent nucleoli accompanied by perinucleolar clearing.^7^ The presence of these features within a morphologically high-grade, heterogeneous tumor can serve as valuable diagnostic clues suggestive of *TFEB*-amplified RCC. The other striking feature of this lesion was the presence of numerous multinucleated tumor giant cells. To our knowledge, this characteristic has not been reported in *TFEB*-amplified RCC and further investigation is required to verify its significance.

*TFEB*-amplified RCC lack pathognomonic histologic features and adequate evaluation requires a broad differential diagnosis. The papillary and pseudopapillary architecture that we observed raised consideration of papillary RCC. This diagnostic challenge is featured in a TCGA sequencing study by Linehan et al, which identified 3 *TFEB*-amplified RCCs among 291 tumors originally classified as papillary RCC.^11^ The expression of melanocytic markers HMB45 and Melan-A is useful in ruling out this possibility, although *TFEB*-amplified RCC frequently lacks HMB45 positivity (as seen in this tumor),^7^ which necessitates the employment of multiple melanocytic markers (usually Mel-A, HMB45, and Cathepsin K) to avoid misclassification.

The tumor's papillary architecture, abundant eosinophilic cytoplasm, and macronucleoli with perinucleolar clearing also overlap with FH deficient RCC. FH deficient RCC is an aggressive tumor often linked to germline mutations and requires genetic counseling. Exclusion of this diagnosis relies on negative 2SC IHC and retention of FH expression.^12^

The presence of polygonal clear/eosinophilic cells arranged in alveolar nests raises consideration of clear cell renal cell carcinoma (CCRCC), a diagnostic challenge further complicated by absence of keratin 7 expression. However, the expression of melanocytic markers strongly argues against this diagnosis, given that these markers are not typically associated with CCRCC.

The other important differential diagnosis for *TFEB*-amplified RCC is malignant epithelioid angiomyolipoma (meAML). The distinction between these two entities can be challenging due to their overlapping immunoprofiles, including consistent expression of melanocytic markers (HMB45/Melan-A), cathepsin K, and often reactivity for KIT and vimentin. While PAX8 is typically retained in *TFEB*-amplified RCCs^7^ and serves as a critical discriminator from PAX8-negative meAML,^13^ the current tumor exhibited only focal weak PAX8 expression which complicated the diagnosis. Although PAX8 loss has been reported in rare *TFEB*-rearranged RCCs,^12^ this phenomenon has not been documented in *TFEB*-amplified RCCs. The diagnostic uncertainty underscores the necessity of molecular confirmation, which was ultimately resolved by FISH analysis confirming *TFEB* amplification—a genetic event not reported in meAML.

*TFEB*-amplified RCCs represent an aggressive RCC with a 5-year cancer-specific survival of 44%-50%.^5,6^ The pathogenesis of this tumor is driven by 6p21.1 locus amplification, which encompasses both *TFEB* and *VEGFA*.^11^ The resultant *VEGF* overexpression renders these tumors potential candidates for anti-angiogenic therapies such as axitinib or pazopanib. Furthermore, PD-L1 expression is reported in these tumors across small cohorts.^14^ This potential therapeutic promise of immunotherapy is highlighted by one documented instance of metastatic *TFEB*-amplified RCC that achieved complete response to anti-PD-1/PD-L1 therapy.^15^ Despite this, predictive biomarkers for immunotherapy remain unclear and mismatch repair (MMR) status in *TFEB*-amplified RCC is poorly characterized. Our tumor retained IHC expression of MSH2, MSH6, MLH1, and PMS2. One prior *TFEB*-amplified RCC reported MSH6 loss by IHC with a coexisting MSH6 missense mutation. However, functional MMR capacity may persist in such circumstances and microsatellite instability was absent.^4^ These findings suggest that the mutation did not fully disrupt DNA repair. In the current tumor, the cells exhibited PD-L1 positivity and the patient was treated with combined immunotherapy and anti-angiogenic treatment. Nevertheless, the patient succumbed to disease progression within 5 months post-surgery. This highlights the therapeutic challenges that can be associated with *TFEB*-amplified RCCs; tumor cells can show strong PD-L1 expression, which is typically a predictor of immunotherapy benefit, but this does not necessarily translate into favorable treatment outcomes.

*TFEB*-amplified RCC represents a new and molecularly distinct RCC subtype with distinct clinicopathologic features. Pathologists should consider a diagnosis of *TFEB*-amplified RCC when encountering a high-grade renal tumor with heterogeneous morphology. Evaluation with melanocytic markers and FISH testing for *TFEB* amplification should be employed to ensure appropriate classification. Accurate diagnosis is essential to guide therapeutic decisions, including the possible roles of immunotherapy and anti-angiogenic treatment, although further investigation is warranted to evaluate the effectiveness of these treatment modalities.
